# Neural Correlates of Personality Traits in Adolescents Exhibiting Excessive Smartphone Use: A Resting-State FMRI Study

**DOI:** 10.3390/life15121899

**Published:** 2025-12-12

**Authors:** Min Kyung Hu, Kyeong Seob Song, Jihye Choi, Arom Pyeon, Hyun Cho, Jung-Seok Choi, Inyoung Choi, Ji-won Chun, Dai-Jin Kim

**Affiliations:** 1 Department of Biomedicine & Health Sciences, College of Medicine, Catholic University of Korea, Seoul 06591, Republic of Korea; helen93@catholic.ac.kr; 2 Department of Psychiatry, Seoul St. Mary’s Hospital, College of Medicine, Catholic University of Korea, Seoul 06591, Republic of Korea; jihye.choi3@gmail.com (J.C.); arom88@hanmail.net (A.P.); sonap1@daum.net (H.C.); 3 Department of Medical Informatics, College of Medicine, Catholic University of Korea, Seoul 06591, Republic of Korea; maze05@nate.com (K.S.S.); iychoi@catholic.ac.kr (I.C.); 4 Department of Psychiatry, Samsung Medical Center, School of Medicine, Sungkyunkwan University, Seoul 06351, Republic of Korea; choijs73@gmail.com; 5 Catholic Medical Center Institute for Basic Medical Science, Catholic Medical Center, Catholic University of Korea, Seoul 06591, Republic of Korea

**Keywords:** excessive smartphone use, adolescence, resting state functional connectivity, persistence, mid cingulate cortex, insula

## Abstract

**Background:** Although smartphone usage is inevitable and convenient in recent days, numerous potential problems due to excessive smartphone use (ESU) have been highlighted. With the rising concern about ESU, the focus on exploring the relationship between ESU and personality traits and their neural correlations also increased; however, studies that explore these factors simultaneously are lacking. **Objective:** This study investigated whether altered resting state functional connectivity (rsFC) is related to personality traits in adolescents exhibiting ESU compared to healthy controls (HCs). **Methods:** Thirty-one adolescents exhibiting ESU and 31 HCs (62 adolescents) aged 12–18 years were included in this study. Seed-to-voxel connectivity analysis was used to examine group differences in rsFC in the middle cingulate cortex (MCC) and insula, key parts of the salience network, in relation to personality traits. **Results:** Adolescents exhibiting ESU showed trends toward low persistence and high harm avoidance in terms of personality traits. Additionally, they exhibited enhanced rsFC between the MCC and insula but reduced rsFC between the precentral and postcentral gyri compared with HCs. Notably, increased rsFC between the MCC and insula in the ESU group was negatively correlated with low persistence. **Conclusions:** ESU was associated with low persistence at the uncorrected threshold in terms of personality traits and involved in neuro-functional alterations between the key hubs of the salience network, MCC, insula, and several other brain regions. These findings may provide a neurobiological basis for intervention targeting behavioral addiction in youth. Accordingly, adolescents with low persistence may need tailored education on appropriate and controlled use of smartphones and internet-based technologies.

## 1. Introduction

Smartphone usage has become inescapable in everyday life, increasing drastically over recent decades. By 2023, more than three-quarters of the adults in North America, Europe, and Asia owned smartphones [1]. The pervasive integration of smartphones into daily life has dramatically transformed communication, leisure-time activities, and social interaction not only among adults but also among adolescents. In addition, the use of smartphones has become indispensable in modern life with the emergence of diverse applications that assist people’s daily routines by self-tracking and analyzing personal data, such as personal fitness, workout performance tracking, diet and nutrition advice, health monitoring, and safety management applications [2]. Recent statistics indicate that smartphone use among adolescents in South Korea is alarmingly high, with over 95% of teenagers reporting using smartphones daily by 2023 [3]. This drastic increase in smartphone use has raised concerns about potential problems resulting from excessive smartphone use (ESU). Previous studies have also suggested that excessive mobile phone use is associated with various complications such as sleep disturbances [4,5], decreased physical fitness [6,7], frequent neck or back pain [7,8], eye strains [9] and headache complaints [10,11]. Furthermore, several studies have indicated a strong correlation between ESU and various psychological problems, including anxiety, depression, cognitive-emotional regulation, and attention-related problems [12,13,14].

Addiction refers to compulsive engagement in rewarding stimuli despite potential adverse consequences that lead to significant impairments [15]. ESU is similar to substance and behavioral addictions, such as Internet gaming disorder, gambling disorder, shopping addiction, and alcohol or drug addiction, as outlined in the DSM-5 [16]. Although the DSM-5 has not yet incorporated ESU as an official behavioral addiction measure, the relevance of ESU to mental health, particularly among adolescents, is significant. This is mainly because adolescent brains undergo critical developmental changes, particularly in areas related to decision-making, social cognition, self-control, impulse control, and reasoning [17]. Previous neuroimaging studies related to ESU revealed the structural and functional brain changes present in individuals with such problematic behaviors [18,19,20,21,22,23,24].

With increasing attention being paid to ESU, researchers have focused on the relationship between ESU and personality traits. In order to examine the personality traits, the Temperament and Character Inventory (TCI) is widely used. TCI comprises four temperament subscales (Novelty Seeking, Harm Avoidance, Reward Dependence, and Persistence) and three personality subscales (Self-Directedness, Cooperativeness, and Self-Transcendence). Previous studies suggest that individuals with ESU exhibit higher levels of novelty seeking and harm avoidance among personality traits, in which novelty seeking is linked to qualities such as disorderliness and impulsivity, and harm avoidance is related to feelings of fear regarding uncertainty and shyness in social interactions [25,26]. Additionally, previous studies have shown that individuals with ESU display higher levels of self-transcendence and lower levels of persistence [25]. Among these previous findings, higher levels of harm avoidance and lower levels of persistence in people with ESU appear to be important personality traits. As persistence is related to achieving goals despite challenges [27,28] and harm avoidance refers to a tendency to fear uncertainty due to worry and anxiety [27,29], individuals with low persistence and high harm avoidance are more prone to using smartphones as a form of escaping from negative emotions, like anxiety [29,30], and from challenges related to achieving tasks and goals in real life [31,32] compared to individuals without these personality traits. Thus, individuals with low persistence and high harm avoidance may struggle to regulate smartphone use, which can lead to ESU. Despite the increase in psychological research on the relationship between personality traits and ESU, neuroimaging studies examining this relationship are scarce.

The Salience Network (SN), which consists of the mid cingulate cortex (MCC) and insula as the central hub, is vital for transitioning between different cognitive states, such as concentrating on external stimuli or engaging in introspective thoughts [33,34,35]. Previous studies indicate that individuals with ESU or problematic internet use exhibit heightened resting-state functional connectivity (rsFC) within the SN [36,37,38] and between the SN and the default mode network (DMN) [36,39]. The DMN, which comprises the medial prefrontal cortex, posterior cingulate cortex, and precuneus, is involved with stimulus-independent and introspective thought processes [40,41]. Furthermore, findings have shown that the SN can influence personality traits related to social behavior, emotional regulation, and impulsivity because of its role in detecting and responding to emotionally salient stimuli [35,42]. Thus, the SN might be strongly related to ESU personality vulnerability. Previous findings have demonstrated that the MCC influences personality traits related to emotional regulation and goal-directed behavior [43,44,45]. Another main region of the SN, the insula, showed increased FC in the right putamen and other brain regions in prolonged bedtime smartphone users [20].

Despite the relevance of exploring the relationship between ESU and personality traits and their neural correlations, existing literature has a dearth of fMRI studies that explore these factors simultaneously. Consequently, addressing this gap is essential for comprehending the developmental implications of ESU in the adolescent brain. Therefore, this study aimed to explore whether altered rsFC in the brain is associated with personality traits in adolescents exhibiting ESU compared to healthy controls (HCs). Building on prior research highlighting the role of the MCC and insula in personality traits and ESU [18,20,24,46,47,48], we selected these areas as regions of interest (ROIs). Furthermore, we aimed to investigate additional brain regions that exhibit rsFC with the MCC and insula in the ESU group. We hypothesized that: (1) adolescents exhibiting ESU would show low persistence and high harm avoidance as personality traits; (2) adolescents exhibiting ESU would show altered rsFC within the SN, including the MCC and the insula, and would demonstrate altered rsFC between the MCC and insula and other parts of the brain regions, including other brain network; and (3) altered FC among the MCC, insula, and other brain areas would correlate with persistence and high harm avoidance levels.

## 2. Methods

### 2.1. Study Participants

In this study, 483 adolescents aged 12–18 years old were recruited online. All participants are smartphone users and completed an online self-reported survey containing questions about general background information such as age, sex, and smartphone usage. Among these participants, 35 adolescents were classified as having ESU using the Smartphone Addiction Proneness Scale for Adolescents (SAPS) for Youth Assessment. Subsequently, 37 age-matched adolescents were recruited from the control group, which consisted of adolescents who used smartphones regularly but did not meet ESU criteria in the SAPS scale.

All participants were right-handed, as examined using the Edinburgh Handedness Inventory [49]. In addition, all participants underwent a clinician-administered structured interview from the Korean Kiddie-Schedule for Affective Disorders and Schizophrenia (K-SADS-PL) to screen for a wide range of psychiatric or major medical disorders, including depressive, anxiety, ADHD, borderline, and Intermittent Explosive Disorders. Thus, participants with these major disorders were excluded from this study through clinician-administered K-SADS-PL. One participant in the ESU group and two participants in the HC group were excluded because of depressive, borderline, or Intermittent Explosive Disorders. Furthermore, three participants in the ESU group and four participants in the HC group were excluded because of distorted imaging data. Eventually, 31 adolescents exhibiting ESU (18 males and 13 females, mean age: 15.23 ± 1.67 years) and 31 HCs (20 males and 11 females, mean age: 15.10 ± 1.51 years) were included in the analysis.

### 2.2. Clinical Assessments

#### 2.2.1. Smartphone Addiction Proneness Scale for Adolescents

ESU was measured using the SAPS [50], which is a self-reported scale consisting of 15 items scored on a four-point Likert scale (1 = Not at all to 4 = Always). The reliability test of the S-scale yielded a Cronbach’s alpha of 0.880. This scale consists of four factors: disturbance of adaptive functions, virtual life orientation, withdrawal, and tolerance. Participants were categorized into the ESU group if their total SAPS score exceeded 42, or if their subscale scores for disturbance of adaptive functions, withdrawal, and tolerance surpassed 14, 12, and 13, respectively.

#### 2.2.2. Adolescent Version of Temperament and Character Inventory (JTCI)

The personality traits of the participants were measured using the JTCI, particularly the Korean version of the JTCI. Lyoo et al. (2004) examined the psychometric properties of the Temperament and Character Inventory (TCI) in the Korean population and reported Cronbach’s alpha coefficients ranging from 0.48 to 0.80 for the temperament scales and 0.64 to 0.68 for the character scales [51].

The TCI and JTCI were designed by Cloninger to examine a person’s temperament and character traits. Cloninger proposed a framework to assess the structure and development of personality by including temperament and character dimensions. The JTCI consists of four temperament subscales (Novelty Seeking, Harm Avoidance, Reward Dependence, Persistence) and three personality subscales (Self-Directedness, Cooperativeness, Self-Transcendence). Subsequently, Cloninger proposed a framework to assess the structure and development of personality by including temperament and character dimensions.

Temperament, characterized by four subscales, is inherited and manifests early in life, involving instinctive responses that contribute to associative learning and habit formation [27,52,53]. We focused on assessing temperament traits, which are foundational components of personality that affect emotional responses and behaviors from an early age, since ESU in adolescents was the main focus of this study.

Harm avoidance indicates individual differences in behavioral inhibition when encountering danger signals or potential punishment [27,52,53]. High harm avoidance is related to shyness toward strangers, pessimism, and worry about the future [27,52,53]. Persistence denotes differences in an individual’s capacity to maintain efforts towards goals despite experiencing fatigue, failure, and frustration [27,52,53]. A person with high persistence is motivated, resilient, and goal-oriented [27,52,53].

### 2.3. Acquisition of Imaging Data

Functional and structural MRI data were acquired using a 3-Tesla MAGNETOM Verio system (Siemens, Erlangen, Germany) with a 16-channel head coil. The participants wore earmuffs for cushioning to reduce head movement. During the scan, the participants were instructed to keep their heads still with their eyes open and focus their eyes on a crosshair. Functional images were captured using a T2*-weighted gradient echo-planar imaging sequence with the following settings: repetition time (TR) = 2000 ms; echo time (TE) = 30 ms; voxel size of 2.0 × 2.0 × 4.0 mm; a matrix size of 96 × 96; and 28 slices. Structural images were captured at a resolution of 1.0 × 1.0 × 1.0 mm using a 3D T1-weighted gradient echo sequence (176 slices, TR = 2300 ms, TE = 2.22 ms, and a 256 × 256 image matrix. The brain fMRI scanning lasted for 400 s, and a total of 200 volumes were collected.

### 2.4. Image Analysis

Resting-state fMRI data were preprocessed and analyzed using the CONN toolbox version 22a (www.nitrc.org/projects/conn, accessed on 24 January 2024) in MATLAB R2022a (MathWorks, Inc., Natick, MA, USA). Functional and anatomical data were preprocessed by removing six initial scans, realignment with correction of susceptibility distortion interactions, outlier detection, slice timing, head motion correction, and normalization to the standard Montreal Neurological Institute space. Anatomical images were then segmented into gray matter, white matter, and cerebrospinal fluid maps. Imaging data were smoothed with a 6 mm Gaussian kernel (full width at half maximum).

Potential outlier scans were identified using the ART scrubbing procedure (https://www.nitrc.org/projects/artifact_detect/, accessed on 24 January 2024) with the 97th percentile in a normative sample (with thresholds for motion = 0.9 mm and global signal z = 5). 6 motion parameters and their first-order derivatives, white matter signal, cerebrospinal fluid signal, and scrubbing were removed through linear regression. Also, the linear trends were removed. The global signal was not regressed out since it can introduce artifactual biases [54], remove potentially meaningful neural components [55], and introduce confounding effects across populations. Then, fMRI data were band-pass filtered at 0.008–0.09 Hz. Additionally, in order to examine group differences in head motion, we compared the mean framewise displacement (FD) across all time points after removing identified outlier scans using an independent samples t-test. The results showed no significant difference between the ESU and HC groups (ESU = 0.11 ± 0.04, HC = 0.12 ± 0.05, t(60) = 0.83, *p* = 0.41, Cohen’s d = 0.21).

We selected the MCC as ROI, which was anatomically defined using the Automated Anatomical Labeling (AAL) atlas. In addition, another ROI insula (left insula region: −32, 24, −10; right insula region: 38, 36, −10) was selected based on a previous study, which focused on internet gaming disorder and personality traits [56] and was defined as a 6 mm-radius sphere centered at the respective coordinates. MCC and insula were selected as ROIs due to their critical roles in the detection and filtration of salient stimuli related to significant environmental events or changes [57,58] and given their strong associations with reward processing, cognitive control, and emotional regulation [18]. These aspects are particularly relevant for understanding personality traits as measured by the temperament subscales of the TCI, which may influence ESU. MCC

Seed-to-voxel FC analysis was used to examine the alteration in functional connectivity related to ESU. Group-level analyses were performed using a General Linear Model (GLM). A separate GLM was estimated with the first-level connectivity measures as dependent variables and groups or other subject-level identifiers as independent variables for each individual connection. Age and sex were included as covariates in the analyses to account for potential confounding variables. The resulting correlation coefficients for each participant were compared using bivariate correlation analysis by group to evaluate between-group differences in seed-to-voxel connectivity. Statistical significance was set at *p* < 0.05, with false discovery rate (FDR) correction. In addition, the cluster threshold was *p* < 0.05, cluster size was *p*-FDR-corrected, and the voxel threshold was *p* < 0.001 *p*-uncorrected.

### 2.5. Statistical Analysis

All statistical analyses associated with demographic and clinical variables, as well as the mean rsFC, were performed using IBM SPSS Statistics for Windows version 24 (IBM Corp., Armonk, NY, USA). A two-sample *t*-test was used to compare group differences in demographic and clinical variables.

The MCC and insula were designated as seed regions, and the reference signal from these seeds was correlated with signals from every voxel throughout the brain to generate FC maps. An Automated Anatomical Labeling map was used to define the ROIs.

Additionally, we conducted a Pearson’s correlation analysis for the ESU and HC groups to examine the relationships between relative rsFC strength and clinical measures, specifically the S-scale and JTCI scores (Supplementary Materials). We also performed a bivariate correlation analysis between the S-scale score and persistence in the total group bivariate correlation analysis and correlation analyses performed by group between the JTCI persistence score and FC z-values for further analyses of specific components of JTCI and rsFC strength.

## 3. Results

### 3.1. Demographic and Clinical Data

The demographic and clinical characteristics of the participants are shown in Table 1. No significant differences were observed between the two groups in terms of sex or age. However, participants with ESU reported significantly higher SAPS scores [*t*(60) = 13.30, FDR corrected *p* < 0.001] compared to HCs. The SAPS subscale scores of disturbance of adaptive functions [*t*(60) = 15.29, FDR corrected *p* < 0.001], withdrawal [*t*(60) = 7.81, FDR corrected *p* < 0.001], and tolerance [*t*(62) = 10.29, FDR corrected *p* < 0.001] were also higher in the ESU group than in the HC group. Additionally, the mean scores for the JTCI subscale of Harm Avoidance [*t*(60) = 2.09, uncorrected *p* < 0.05, FDR corrected *p* = 0.08] were higher in the ESU group than in the HC group, only in the uncorrected *p* value. In contrast, the mean scores of Persistence [*t*(60) = −2.20, uncorrected *p* < 0.05, FDR *p* = 0.08] were lower in the ESU group compared to the HC group, only in the uncorrected p value. The mean Novelty Seeking scores were higher in the ESU group than in the HC group, but the difference was not statistically significant [*t*(60) = 1.72, uncorrected *p* = 0.090, FDR *p* = 0.12]. In addition, the average Reward Dependence scores in the ESU group were higher than those in the HC group, although this group difference was not statistically significant [*t*(60) = 1.28, uncorrected *p* = 0.206, FDR *p* = 0.21].

### 3.2. Alterations in Functional Connectivity

**Figure 1:**
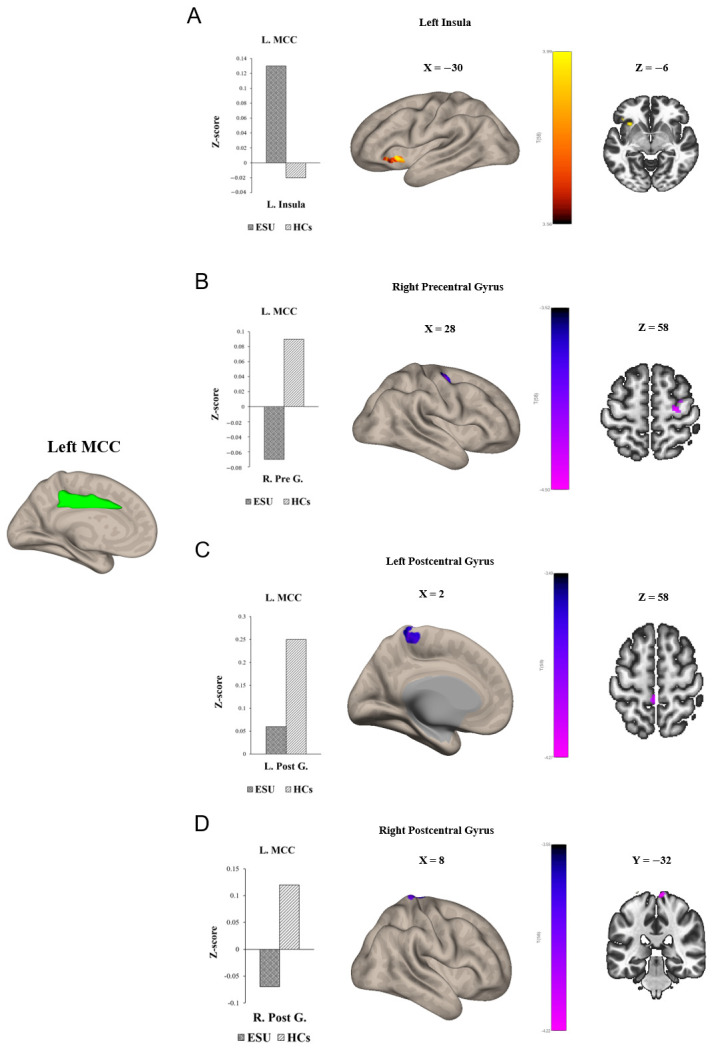
Group differences in seed to voxel functional connectivity. Note: seed ROI is left MCC. The bar graphs illustrate the resting–state functional connectivity strength (rsFC) between (**A**) the left MCC and the left insula and (**B**) the left MCC and the right Precentral Gyrus, (**C**) the left MCC and the left Postcentral Gyrus, and (**D**) the right Postcentral Gyrus. (**A**) shows increased rsFC between the left MCC and the left insula. (**B**) shows decreased rsFC between left MCC and right Precentral Gyrus. (**C**,**D**) show decreased rsFC between the left MCC and both sides of the Postcentral Gyrus. ESU = excessive smartphone use; HCs = healthy controls; L. MCC = left mid cingulate cortex; L. Insula = left insula; R. Pre G. = right Precentral Gyrus; L. Post G. = left Postcentral Gyrus; R. Post G. = right Postcentral Gyrus.

**Figure 2:**
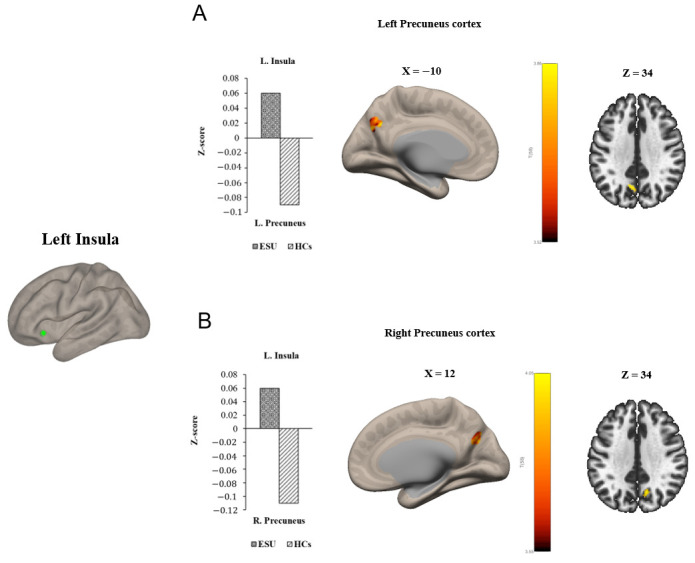
Group differences in seed to voxel functional connectivity. Note: Seed ROI is left insula. The bar graphs illustrate the resting-state functional connectivity strength (rsFC) between (**A**) the left insula and the left Precuneus Cortex and (**B**) the left insula and the right Precuneus Cortex. (**A**) and (**B**) show increased rsFC between left insula and both sides of Precuneus Cortex. ESU = excessive smartphone use; HCs = healthy controls; L. Insula = left insula; L. Precuneus = left Precuneus Cortex; R. Precuneus = right Precuneus Cortex.

Group differences in seed-to-voxel FC are illustrated in Figure 1 and Figure 2 and Table 2. In particular, the ESU group showed higher FC between the left MCC (L. MCC) and left insular cortex (L. Insula) [*t*(58) = 5.83, corrected *p* < 0.05; Figure 1A] compared to the HC group. In contrast, the ESU group showed weakened connectivity between the L. MCC and right precentral gyrus (R. Precentral Gyrus) [*t*(58) = 5.42, corrected *p* < 0.05; Figure 1B], and between the L. MCC and Right Postcentral gyrus (R. Postcentral Gyrus) [*t*(58) = 4.87, corrected *p* < 0.05; Figure 1D] and Left Postcentral Gyrus (L. Postcentral Gyrus) [*t*(58) = 5.36, corrected *p* < 0.05; Figure 1C] compared to the HC group. Furthermore, the ESU group showed high FC between the L. Insula and both sides of the precuneus cortex: Right Precuneus Cortex (R. Precuneus) [*t*(58) = 4.22, corrected *p* < 0.05; Figure 2B] and Left Precuneus Cortex (L. Precuneus) [*t*(58) = 4.26, corrected *p* < 0.05; Figure 2A]. However, the Right MCC (R. MCC) and right insular cortex (R. Insula) did not show significant group differences in seed-to-voxel FC with other brain regions.

### 3.3. Correlation Between Functional Connectivity and JTCI: Persistence

**Figure 3:**
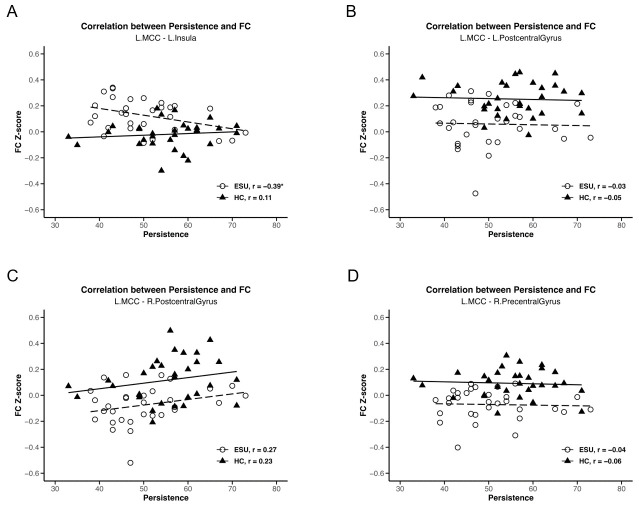
Correlations between MCC rsFC strength and TCI persistence score. Negative correlation between (**A**) L. MCC/L. Insula and persistence (r = −0.39, *p* < 0.05). (**B**) L. MCC/L. Postcentral Gyrus and persistence, (**C**) L. MCC/R. Postcentral Gyrus and persistence and (**D**) L. MCC/R. Precentral Gyrus and persistence do not show a statistically significant correlation. * *p* < 0.05, ESU = excessive smartphone use; HC = healthy control; FC = functional connectivity; L. = left; R. = right.

We conducted a separate-group correlation analysis between rsFC strength and two subscales of the JTCI, Harm Avoidance and Persistence, which showed statistically significant group differences. As shown in Figure 3A, a negative correlation was observed between L. MCC-L. Insula rsFC strength and JTCI persistence scores (r = −0.39, *p* < 0.05), which were higher in the ESU group than in the HC group. This outcome established that the increased connectivity between the L. MCC and L. Insula correlated with lower persistence in adolescents exhibiting ESU. However, the rsFC strength of the L. MCC-R. Precentral Gyrus (Figure 3B), the L. MCC-R. Postcentral Gyrus (Figure 3D) and the L. MCC-L. Postcentral Gyrus (Figure 3C) and JTCI Persistence scores did not demonstrate a significant correlation. The L. MCC rsFCs did not show a statistically significant correlation with the JTCI Harm Avoidance score. Additionally, L. insula-R. Precuneus, and L. Insula-L. Precuneus rsFC strength did not exhibit a statistically significant correlation with either JTCI persistence or harm avoidance.

## 4. Discussion

### 4.1. Principal Findings and Comparison with Previous Works

The current study is among the first to examine the relationship among ESU, personality traits, and alterations in the rsFC of the SN, focusing on the MCC and insula in adolescents exhibiting ESU. Previous studies have examined the relationship between ESU and personality traits, the relationship between personality traits and alterations in the rsFC of brain regions, and the changes in rsFC in individuals with ESU in separate dimensions. This present study is distinctive in that it integrates all these relationships regarding ESU. In this study, adolescents exhibiting ESU showed low persistence and high harm avoidance. Additionally, adolescents exhibiting ESU exhibited alterations in the rsFC of the L. MCC and L. Insula. Furthermore, L. MCC-L. Insula rsFC strength was correlated with persistent temperament traits in adolescents exhibiting ESU.

Our findings illustrated that adolescents exhibiting ESU exhibited lower levels of persistence and higher levels of harm avoidance than the HC group. This highlights the critical relationship between these personality traits and the tendency to develop problematic ESU behaviors. Adolescents exhibiting ESU and low persistence could be vulnerable to the instant pleasure provided by using smartphones and may experience difficulty maintaining long-term focus. This tendency could reinforce behavioral addiction-like ESU cycles. This is consistent with previous studies, which claimed a relationship between ESU individuals and low persistence [25] and suggested a potential connection between addictive behaviors of ESU and difficulties in goal-directed behavior [31]. Additionally, we found that high harm avoidance, a personality trait closely related to anxiety and worry, was associated with ESU risk. Thus, adolescents exhibiting ESU and high harm avoidance may use smartphones as a coping mechanism to manage the negative emotions caused by anxiety, effectively utilizing their devices to escape uncomfortable feelings. This finding is in line with previous studies, which showed a significant correlation between high harm avoidance and increased risk of ESU [29,59], indicating that these individuals may use smartphones to avoid anxiety existing in real life. In addition, previous research has claimed that low persistence and high harm avoidance were associated with internet addiction and gambling disorder [60]. Accordingly, our findings show that ESU demonstrates the same personality characteristics as behavioral addictions. Moreover, our results suggest that enhancing persistence and regulating harm avoidance could benefit adolescents exhibiting ESU by inhibiting their vicious addictive behavioral cycle.

In this study, adolescents exhibiting ESU demonstrated increased levels of rsFC between the L. MCC and the L. Insula. The critical association between the SN and cognitive-emotional dynamics related to ESU was illustrated by the elevated rsFC strength of the L. MCC-L. Insula. Additionally, heightened connectivity reflected enhanced sensitivity to salient stimuli [61], particularly those associated with smartphone interactions, such as phone notifications. The insula is critical in the decision-making and processing of emotional salience, integrating sensory information to guide behavioral responses [20,62,63,64]. In addition, the insula plays a significant role in behavioral and substance addiction, like internet gaming disorder and drug addiction [65,66]. Along with the insula, the MCC is essential for emotional processing and cognitive control [67]. Thus, the increased rsFC of the MCC-insula in adolescents exhibiting ESU suggested that they would experience difficulty in regulating ESU. This is mainly because increased connectivity of the MCC-insula in adolescents exhibiting ESU could lead the brain network to lose cognitive control over smartphone use and be more attentive to smartphone-related external stimuli and rewards provided by smartphones, such as smartphone notification alarms, online games, and social media. Our findings align with those of previous studies that documented similar patterns, indicating that individuals with ESU often demonstrated heightened FC within the SN [36,68,69,70]. We found enhanced connectivity between the MCC and insula in adolescents exhibiting ESU, highlighting the potential difficulties in impulse control, cognitive flexibility, and reinforcement of addictive behaviors [71,72]. Therefore, hyper-connectivity of the MCC in the insula could be an important neuro-biomarker of ESU.

Our rsFC analysis revealed a hyper-connectivity between the insula and both sides of the precuneus in adolescents exhibiting ESU. Increased connectivity between the L. Insula, a part of the SN involved in processing emotional salience and decision-making, and the precuneus, a core region of the DMN involved in self-referential and introspective thoughts [73,74], highlights decreased engagement of the executive control and reflective system [36,75]. Aligned with our findings, the previous study showed the increased connectivity of insula-precuneus in participants with ESU [36]. Consequently, the hyper-connectivity of the insula-precuneus in adolescents exhibiting ESU could influence them to struggle with controlling smartphone use and maintain ESU behaviors.

Moreover, in this study, adolescents exhibiting ESU presented a weakened rsFC between the L. MCC and R. precentral gyrus, and between the L. MCC and both sides of the postcentral gyrus. The reduced connectivity between the left MCC and right precentral gyrus, which is part of the primary motor cortex responsible for coordinating voluntary movements [76], may imply that adolescents exhibiting ESU may experience difficulties in initiating and controlling their behaviors. However, prior research on alterations in the precentral gyrus was scarce. Additionally, the postcentral gyrus is part of the Primary Somatosensory Cortex and is involved in processing sensory information. Thus, the decreased connectivity between the L. MCC and postcentral gyrus indicates that adolescents exhibiting ESU may face challenges in integrating sensory input to control smartphone use. Thus, these alterations in the precentral gyrus and postcentral gyrus suggest potential impairments in the interaction between behavioral regulation and sensory processing in adolescents exhibiting ESU.

A negative correlation between increased L. MCC-L. Insula rsFC strength and low persistence score in adolescents exhibiting ESU were observed in the present study. This finding highlights a significant relationship between FC and personality traits. Thus, our findings indicate that adolescents exhibiting ESU with increased connectivity in the MCC-insula and low persistence may struggle more to control and overcome the addictive behaviors of ESU than those who do not have these features. In addition, these findings emphasize that low persistence can inhibit the ability to regulate ESU in adolescents. Thus, adolescents exhibiting ESU exhibiting lower persistence may be more likely to yield to the immediate gratifications offered by smartphone use, resulting from heightened sensitivity to emotionally salient stimuli processed by the SN, which could lead them to prioritize smartphone use over goal-directed activities. Thus, the interaction between enhanced rsFC in the MCC-insula and low persistence may reinforce addiction to smartphone use in Adolescents exhibiting ESU. These findings suggest that the interplay between the SN and persistence is vital for understanding the cognitive and emotional challenges related to addictive behaviors faced by adolescents exhibiting ESU. In addition, developing interventions for ESU that include improving persistence would be pivotal in helping adolescents exhibiting ESU overcome their addictive behaviors.

As mentioned in the results, left-lateralized alterations of SN were acknowledged in this study since R. MCC and R. Insula did not show significant group differences in seed-to-voxel FC with other brain regions. Notably, several previous investigations examining SN alterations in alcohol use disorder and problematic smartphone use have also reported changes localized mainly within the left SN [36,77]. Therefore, these findings suggest that individuals with ESU or those involved in behavioral and substance addictions may exhibit left-lateralized alterations in the SN.

As shown in this study, ESU is associated with altered rsFC within the SN and its connections to other brain regions, a pattern that mirrors findings in behavioral [78] and substance addictions such as internet gaming disorder [68,79] and nicotine dependence [80]. Thus, ESU appears to exhibit neural alteration patterns highly similar to those observed in these addictive conditions, suggesting a close neurobiological relationship between ESU and other forms of addiction. Moreover, the association between SN dysfunction and personality traits identified in ESU also aligns with patterns reported in behavioral and substance addictions. Prior studies in internet gaming disorder and cocaine addiction indicated that disrupted integration of internal and external stimuli within the SN was linked to maladaptive emotional responses [81], impulsivity, and reward sensitivity [63]. Consistent with these findings, the present study demonstrates that SN alterations in ESU are related to personality traits relevant to emotional regulation, particularly low persistence, which supports shared vulnerability mechanisms across ESU and other addictive behaviors.

### 4.2. Future Directions

Since a relationship between hyper-connectivity within the SN and low persistence was observed in adolescents exhibiting ESU, targeted interventions focusing on enhancing cognitive control, emotional regulation, and persistence are required. One possible intervention is cognitive behavioral therapy (CBT) with mindfulness techniques that address persistence-enhancing skills and emotion regulation strategies, as CBT is a renowned intervention for behavioral and substance addictions. CBT improves self-regulation and reduces reliance on maladaptive coping mechanisms related to behavioral addiction, such as pathological gambling, Internet addiction, and ESU [80,82,83]. In addition, schools can create structured programs that encourage real-life social interactions and intriguing activities requiring sustained focus, such as sports, arts, musical instruments, and community service clubs. Such initiatives could effectively diminish the temptation to use smartphones while fostering persistence in achieving personal and academic goals in adolescents exhibiting ESU. Another innovative approach is transcranial direct current stimulation (tDCS), a non-invasive neuro-modulation technique that can alter diffused rsFC [79,84]. Previous studies have indicated that tDCS can increase or decrease the distorted rsFC between brain regions involved in cognitive control and emotional regulation [81,85,86,87]. Thus, tDCS could be used to reduce and modulate hyperactivity in the MCC and insula, which may in turn alleviate impulsive behaviors associated with ESU. Consequently, applying tDCS to MCC and insula, which are involved in emotion and impulse regulation, may improve cognitive control and reduce sensitivity to smartphone-related stimuli in adolescents.

Based on the findings from this study, future research should focus on developing experimental designs or intervention studies related to enhancing persistence and ESU to examine whether persistence improvement plays a role in reducing engagement with smartphone use. Moreover, exploring the interaction effects with other personality traits and altered rsFC in different brain regions in people with ESU may yield a more comprehensive understanding of individual differences and neurobiological factors in response to ESU.

## 5. Limitations

Nonetheless, the present study has some limitations. Firstly, the sample size was relatively small (n = 62), and data were collected at a single site. Thus, future research with larger multi-site samples is needed to enhance the generalizability of the findings to the broader adolescent population. Secondly, the current study utilized cross-sectional data, which limited the ability to infer causal relationships between ESU and personality traits and altered rsFC in the SN. Therefore, further longitudinal fMRI studies are required to explore the interactions and causation of these variables over time. Thirdly, a report of daily smartphone use metrics, such as smartphone use duration, was not collected in this study, so further studies should collect smartphone use duration data for convergent validity of this study. Finally, other limitations of this study are limited coverage, self-report measures, and potential residual motion effects, which should be considered in future research.

## 6. Conclusions

The findings of this study provide evidence that adolescents exhibiting ESU demonstrate several altered FCs in the SN, which comprises the MCC and insula. Particularly, hyper-connectivity between the MCC and insula indicated dysregulated connectivity within the SN, whereas hyper-connectivity between the insula and precuneus illustrated dysregulated connectivity between the SN and DMN in adolescents exhibiting ESU. In contrast, adolescents exhibiting ESU exhibited weakened rsFC between the MCC and the precentral and postcentral gyri, which demonstrated impaired connectivity between the SN and the primary motor and somatosensory cortices. Furthermore, dysregulated hyper-connectivity within the SN was correlated with lower persistence in adolescents exhibiting ESU. Consequently, these findings pave the way for discovering possible ways to enhance persistence and cooperate with useful persistence-enhancing skills in ESU interventions to control ESU symptoms.

## Figures and Tables

**Table 1:** Demographic characteristics of participants.

	ESU (n = 31)		HC (n = 31)		t-Score	Uncorrected *p*	FDR Corrected *p*	Cohen’s d
	Mean	SD	Mean	SD				
Age	15.23	1.668	15.1	1.513	0.32	0.75	0.75	0.081
Gender								
Male	58.1% (n = 18)		64.5% (n = 20)		χ2 = 0.27			
Female	41.9% (n = 13)		35.5% (n = 11)					
SAPS	44.06	6.48	24.32	5.13	13.30 **	<0.001	<0.001	3.38
Disturbance of adaptive function	15.52	1.46	8.26	2.21	15.29 **	<0.001	<0.001	3.88
Withdrawal	10.65	2.88	6.03	1.58	7.81 **	<0.001	<0.001	1.98
Tolerance	13.35	2.12	7.58	2.29	10.29 **	<0.001	<0.001	2.62
JTCI								
Novelty seeking (NS)	46.77	9.52	43.06	7.29	1.72	0.09	0.12	0.44
Harm avoidance (HA)	50.19	10.21	44.81	10.11	2.09 *	0.04	0.08	0.53
Reward Dependence (RD)	51.71	8.84	48.71	9.62	1.28	0.21	0.21	0.32
Persistence (P)	50.19	9.15	55.29	9.06	−2.20 *	0.03	0.08	−0.56

* Uncorrected *p* < 0.05, ** FDR corrected *p* < 0.001. ESU, excessive smartphone use; HC, healthy control; SAPS, Smartphone Addiction Proneness Scale; JTCI, adolescent version of temperament and character inventory.

**Table 2:** Seed to voxel functional connectivity.

Seed	Regions	Peak MNI (mm)			T-Value	Voxels	Group Difference	*p*-FDR	$\eta_{p}^{2}$
		X	Y	Z					
**L. MCC**									
	L. Insula	−30	18	−04	5.83	66	ESU>HC	<0.001	0.33
	R. Precentral Gyrus	28	−14	58	5.42	195	ESU<HC	<0.001	0.37
	L. Postcentral Gyrus	02	−36	60	5.36	208	ESU<HC	<0.001	0.33
	R. Postcentral Gyrus	08	−32	76	4.87	95	ESU<HC	<0.001	0.29
**R. MCC**	None								
**L. Insula**									
	R. Precuneus	12	−66	32	4.22	80	ESU>HC	<0.001	0.23
	L. Precuneus	−10	−62	32	4.26	73	ESU>HC	<0.001	0.24
**R. Insula**	None								

FDR-corrected *p* < 0.05. ESU, excessive smartphone use; HC, healthy control; L., left; R., right; MCC, Mid Cingulate Cortex.

## Data Availability

The datasets used during this study are available from the corresponding author upon reasonable request.

## Supplementary Materials

The following supporting information can be downloaded at: https://www.mdpi.com/article/10.3390/life15121899/s1, Table S1: Results of Correlation Analysis, Excel S1: Functional Connectivity Data Table.
