# Supplementary material for: Neural Correlates of Personality Traits in Adolescents Exhibiting Excessive Smartphone Use: A Resting-State FMRI Study
Source: Life (Basel). 2025 Dec 12;15(12):1899. doi: 10.3390/life15121899 (PMC12734216; doi:10.3390/life15121899)
Supplement: Supplementary file 1 [file life-15-01899-s001.zip › Supplementary Table S1.pdf]

Supplementary Table S1. Results of Correlation Analysis

		ESU(n=31)		HC(n=31)	
**JTCI**	**rsFC regions**	**r**	**p**	**r**	**p**
**Harm Avoidance(HA)**	L. MCC, L. Insula	0.10	0.60	0.02	0.91
	L. MCC, R. Precentral Gyrus	0.21	0.26	0.04	0.82
	L. MCC, L. Postcentral Gyrus	0.21	0.25	0.18	0.33
	L. MCC, R. Postcentral Gyrus	0.27	0.15	0.14	0.45
	L. Insula, R. Precuneuous	0.09	0.62	0.07	0.70
	L. Insula, L. Precuneuous	0.17	0.37	0.15	0.42
**Persistence(P)**	L. MCC, L. Insula	-0.39	0.03	0.11	0.55
	L. MCC, R. Precentral Gyrus	-0.04	0.83	-0.06	0.73
	L. MCC, L. Postcentral Gyrus	-0.03	0.86	-0.05	0.79
	L. MCC, R. Postcentral Gyrus	0.27	0.14	0.23	0.22
	L. Insula, R. Precuneuous	-0.19	0.31	-0.08	0.68
	L. Insula, L. Precuneuous	-0.33	0.07	0.01	0.97
